# Supplementary material for: Top-down, bottom-up, and history-driven processing of multisensory attentional cues in intellectual disability: An experimental study in virtual reality
Source: PLoS One. 2021 Dec 21;16(12):e0261298. doi: 10.1371/journal.pone.0261298 (PMC8691646; doi:10.1371/journal.pone.0261298)
Supplement: S1 File — (DOCX) [file pone.0261298.s001.docx]

**Supplementary analyses on the speed-accuracy tradeoff and log-transformed RTs**

1. Examination of the speed-accuracy tradeoff and gender effect

Means of response time (RT) and accuracy rate in all cue types are reported separately for the TD and ID group in Table 1 and 2, respectively. Since neutral cues were identical in terms of containing *no information* about the location of the upcoming target, we first checked whether there were significant differences in RT or accuracy rate between neutral cue types by conducting mixed ANOVAs with neutral cue type (visual, auditory, tactile) as a within-subject factor and group as a between subject factor. The results revealed no significant main effect of neutral cue type [RT: *F*(2, 76) = 1.945, *p =* .150, $\eta_{p}^{2}$ = .049 ; Accuracy rate: *F*(2, 76) = 1.373, *p =* .260, $\eta_{p}^{2}$ = .035] or interaction between neutral cue type and group [RT: *F*(2, 76) = .462, *p =* .632, $\eta_{p}^{2}$ = .012; Accuracy rate: *F*(2, 76) = .725, *p =* .488, $\eta_{p}^{2}$ = .019]. Thus, search performance from neutral cue trials were collapsed in subsequent analyses.

**Table 1. Mean RT (ms) and accuracy rate (in parenthesis) in each cue type condition in the TD group**

|  | **Visual** | | **Auditory** | | **Tactile** | |
| --- | --- | --- | --- | --- | --- | --- |
| **Endogenous** | 812 (1.00) | | 841 (.995) | | 838 (.998) | |
| **Exogenous** | Color | Motion | Verbal | Non-verb | HF | LF |
|  | 823 (.998) | 831 (1.00) | 792 (1.00) | 790 (1.00) | 807  (.992) | 800 (.998) |
| **Neutral** | 1270 (.983) | | 1316 (.992) | | 1237 (.992) | |

Table 2. Mean RT (ms) and accuracy rate (in parenthesis) in each cue type condition in the ID group

|  | **Visual** | | **Auditory** | | **Tactile** | |
| --- | --- | --- | --- | --- | --- | --- |
| **Endogenous** | 1440 (.985) | | 1700 (.965) | | 1698 (.980) | |
| **Exogenous** | Color | Motion | Verbal | Non-verb | HF | LF |
|  | 1613 (.998) | 1445  (.983) | 1364  (.968) | 1427  (.985) | 1400  (.978) | 1422  (.990) |
| **Neutral** | 1855 (.945) | | 1840 (1.00) | | 1. (.994) | |

We next examined whether there were effects involving cue type and group in RT and accuracy rate, separately. A mixed ANOVA on the RT data with cue type as a within-subject factor and group as a between subject factor revealed a significant main effect of group [*F*(1, 38) = 54.70, *p <* .001, $\eta_{p}^{2}$ = .590], with a slower mean RT in the ID group (1527 ms) than in the TD group (857 ms). The main effect of cue type [*F*(9, 342) = 25.75, *p <* .001, $\eta_{p}^{2}$ = .404] and the interaction between cue type and group [*F*(9, 342) = 5.85, *p <* .001, $\eta_{p}^{2}$ = .133] were also significant. A mixed ANOVA on the accuracy rate data with cue type as a within-subject factor and group as a between subject factor revealed a significant main effect of group [*F*(1, 38) = 7.39, *p =* .01, $\eta_{p}^{2}$ = .163], with a higher mean accuracy rate in the TD group (.997) than in the ID group (.982). The main effect of cue type [*F*(9, 342) = 1.96, *p =* .04, $\eta_{p}^{2}$= .049] and the interaction between cue type and group [*F*(9, 342) = 2.21, *p =* .02, $\eta_{p}^{2}$= .055] were also significant.

In order to examine whether there was the speed-accuracy tradeoff or not, we drew the speed-accuracy tradeoff function (Heitz, 2014), which plotted mean RT and accuracy rate for each cue type condition separately (Fig. 1). The direct relationship was examined by correlating each condition’s mean RT and mean accuracy rate. For the TD group, there was a significant *negative* correlation between RT and accuracy rate (*r* = -.80, *p =* .002), indicating that the conditions with higher mean accuracy rates also tended to have faster mean RTs. This is the *opposite* of the definition of speed-accuracy trade-off. For the ID group, the correlation between RT and accuracy rate was not significant (*r* = .45, *p =* .143), showing no sign of the speed-accuracy tradeoff. Since there were significant effects involving cue type for both RT and accuracy rate, and also there was no evidence of the speed-accuracy tradeoff, inverse efficiency (IE) values (Townsend & Ashby, 1983) were calculated and analyzed for each participant/cue condition to *combine* the effects of RT and accuracy in all subsequent analyses in the main manuscript.


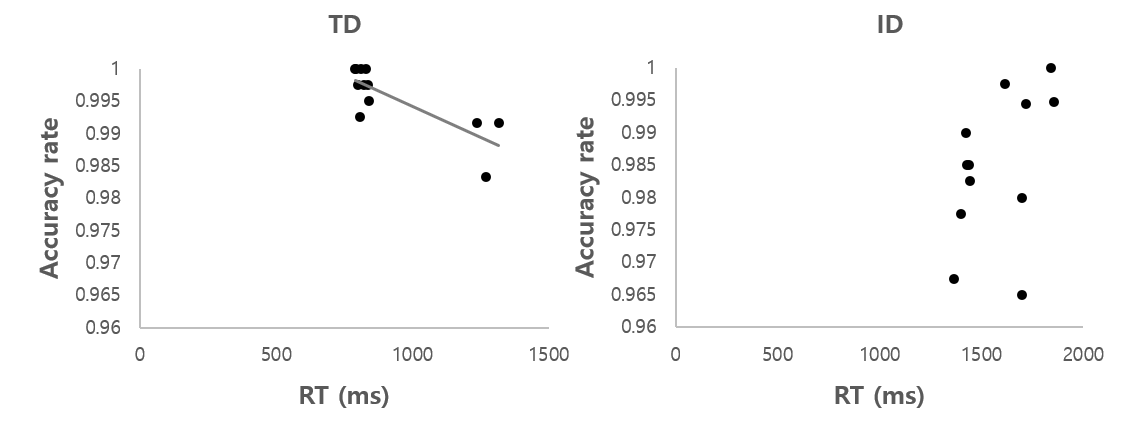


Figure 1. A. a scatter plot (with a best-fitting regression line) illustrating the relationship between RT and accuracy rate in each cue type condition in the TD group. B. a scatter plot illustrating the relationship between RT and accuracy rate in each cue type condition in the ID group.

To examine the possibility that gender of the participants might have differentially influenced attentional performance, we conducted our main analyses again with gender as a between-subject factor; The cue effect data were entered into a mixed ANOVA with sense (visual, auditory, tactile) and origin (endogenous, exogenous) of the cue as within-subject factors and gender (female, male) as a between-subject factor. The results revealed no significant main effect [*F*(1, 38) = .05, *p* = .82, $\eta_{p}^{2}$ = .001] or interactions [*F*s < 1.66, *p*s > .20, $\eta_{p}^{2}$s < .042] involving gender, supporting that gender did not differentially affected the search performance of participants.

1. Analyses on log_10_-transformed RTs

Although we observed no sign of the speed-accuracy tradeoff, it would be reassuring to confirm that analyses based only on RTs reproduce the same pattern of results as in the IE-based analyses. Also, given the much slower RTs in the ID group than in the TD group, it might be more appropriate to analyze log-transformed RTs, in order to make sure that the interaction between group and cue type is not due to the proportional slowing of the RTs in the ID group. Thus, we conducted the same analyses as in the main manuscript on the log-transformed RT data, and verified whether the major effects we observed in the IE-based analyses are replicated or not.

First, the cue effect index was calculated by subtracting the log_10_-transformed RT for each cue type from that of the neutral cue, for each participant. Next, we evaluated the efficiency of top-down and bottom-up processing of multisensory cues by entering the cue effect data into a mixed ANOVA with sense (visual, auditory, tactile) and origin (endogenous, exogenous) of the cue as within-subject factors and group (ID, TD) as a between-subject factor (Fig. 2). The results showed a significant main effects of origin [*F*(1,  38) = 15.76, *p <* .001, $\eta_{p}^{2}$ = .293] and group [*F*(1, 38) = 36.43, *p <* . 001, $\eta_{p}^{2}$ = .489], but no significant main effect of sense (*F*(2, 76) = .59, *p* = .557, $\eta_{p}^{2}$ = .015). Overall, the effect of exogenous cues (.111) was greater than that of endogenous cues (.138), with a higher mean cue effect in the TD group (.182) as compared to the ID group (.067). Importantly, the interaction between origin and group was significant [*F*(1, 38) = 5.73, *p =* .022, $\eta_{p}^{2}$ = .131], indicating that the effect of origin of cues occurred differently in the TD and ID group. Separate repeated measures ANOVAs for the two groups revealed no significant difference in the cue effect between endogenous and exogenous cues in the TD group [*t*(19) = 1.57, *p =* .133, *d* = .351], whereas in the ID group the mean cue effect was significantly larger for exogenous cues (.089) than endogenous cues (.045) [*t*(19) = 3.68, *p =* .002, *d* = .822]. This result supports the hypothesis that the deficits in ID are more pronounced in the top-down attentional mechanism as compared to the bottom-up attention mechanism. Finally, sense by origin interaction [*F*(2, 76) = 17.89, *p <* .001, $\eta_{p}^{2}$ = .320] and sense by origin by group interaction [(*F*(2, 76) = 6.27, *p =* .003, $\eta_{p}^{2}$ = .142] were significant. Separate repeated measures ANOVAs for the two groups revealed that in the TD group, the main effects of sense and origin were not significant (*F*s < 2.47, *ps >* .13, *d*s < .115), but the interaction between sense and origin was significant [*F*(2, 38) = 3.77, *p =* .03, $\eta_{p}^{2}$ = .165]. Post-hoc comparisons with Bonferroni correction showed no significant difference between exogenous and endogenous cues in all sensory modalities [*t(19)*s < 2.51, *ps >* .063, *d*s < .560] in the TD group. In the ID group, the main effect of origin was significant [*F*(1, 19) = 13.53, *p =*  .002, $\eta_{p}^{2}$ = .416], as well as the interaction between sense and origin [*F*(2, 38) = 14.25, *p <* .001, $\eta_{p}^{2}$ = .429]. The post-hoc comparisons with Bonferroni correction showed that exogenous cues were more effective than endogenous cues in both auditory [*t*(19) = 6.60, *p <* .001, *d* = .1.475] and tactile [*t*(19) = 3.79, *p =* .003, *d* = ..847] sensory modalities, but not in visual sensory modality [*t*(19) = 1.25, *p =* .675, *d* = .280] in the ID group.

In summary, all the major effects we observed with the IE data were replicated with the log_10_-transformed RT data. This provides evidence that the observed interaction between group and cue type is not simply an artifact of the perceptual/motor-speed difference between groups, but indeed reflects an effect on attentional guidance.


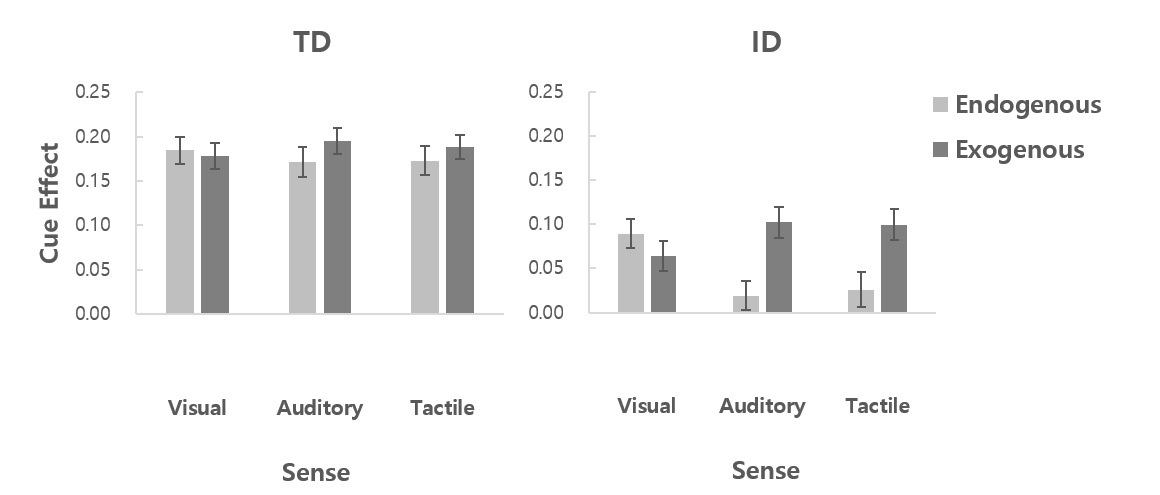


Figure . Mean cue effect for each cue type, based on the origin (endogenous, exogenous) and sense (visual, auditory, tactile) of the cue, shown separately for the TD and ID groups. The cue effect was calculated by subtracting the log_10_-transformed RT value for each cue type from that of the neutral cue, such that higher values represent greater cueing effects.

We also re-examined whether the effects of exogenous cues in guiding attention differed across six feature dimensions with the log_10_-transformed RT data. The cue effect data calculated from log_10_-transformed RTs were entered into a mixed ANOVA with cue type (6 exogenous cues) as a within-subject factor and group as a between-subject factor (Fig 3). The main effect of group was significant [*F*(1, 38) = 24.26, *p <* .001, $\eta_{p}^{2}$ = .390)], with a larger mean cue effect in the TD group (.187) than in the ID group (.090). The main effect of cue type was also significant [*F*(5, 190) = 4.31, *p =* .001, $\eta_{p}^{2}$ = .102)], and pairwise comparisons with Bonferroni-correction indicated larger cue effect for auditory verbal cue (.154) than for visual color cue (.112) [*t*(19) = 3.27, *p =* .03, *d* = .517]. All other pairs did not reach significance. The interaction between group and cue type was not significant (*F*(5, 190) = 2.20, *p =* .056, $\eta_{p}^{2}$ = .055), indicating that the pattern of the cue effect across exogenous cues was similar for both groups. Again, analyses based only on log_10_-transformed RT data reproduced similar pattern of results as in the IE-based analyses, that the effect of exogenous cues to guide attention was distributed rather evenly across feature dimensions for both people with ID and typically developing controls.


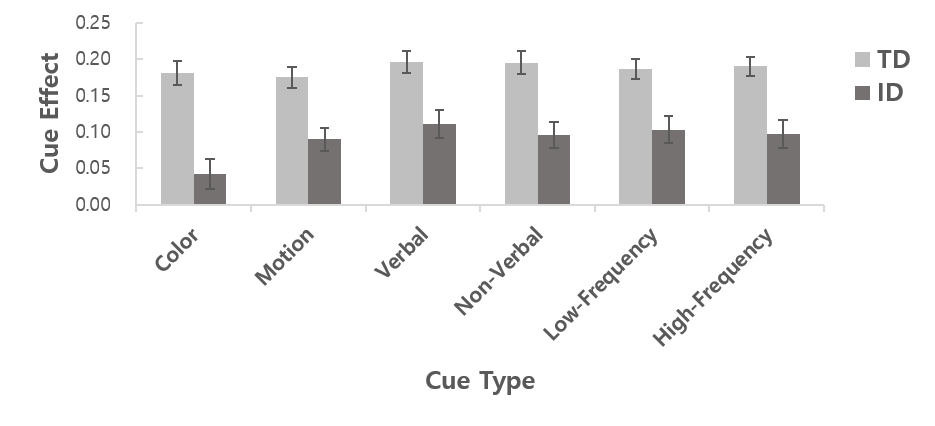


Figure . Mean cue effect for each exogenous cue type, shown separately for the TD and ID groups.

Finally, the efficiency of history-driven attentional mechanism was re-examined with the log_10_-transformed RT data. The log_10_-transformed RT data were entered into a mixed ANOVA with cue type repetition (repeated, non-repeated) as a within-subject factor and group (TD, ID) as a between-subject factor (Fig 4A). There was a significant interaction between cue type repetition and group [*F*(1, 38) = 48.02, *p <* .001, $\eta_{p}^{2}$ = .558], indicating that the pattern of cue type repetition priming was different between groups. Separate paired t-tests for the two groups revealed a significant repetition priming effect in the TD group, with better search performance in cue type-repeated trials (2.900) than in non-repeated trials (2.930) (*t(19)* = 7.14, *p <* .001, d = 1.597). On the contrary, the ID group showed a significant *decrease* in search efficiency in cue type-repeated trials (3.192) as compared to non-repeated trials (3.171) (*t(19)* = 3.49, *p =* .002, *d* = .779): The repetition of the same cue type had an *adverse* effect on search efficiency in the ID group, which was opposite to the pattern observed in the TD group. These results replicate all the major effects we observed with the IE data, and suggest that the function of history-driven attentional mechanism that enhances the processing of *repeated features of stimuli* is not only deteriorated but even reversed in individuals with intellectual disability.


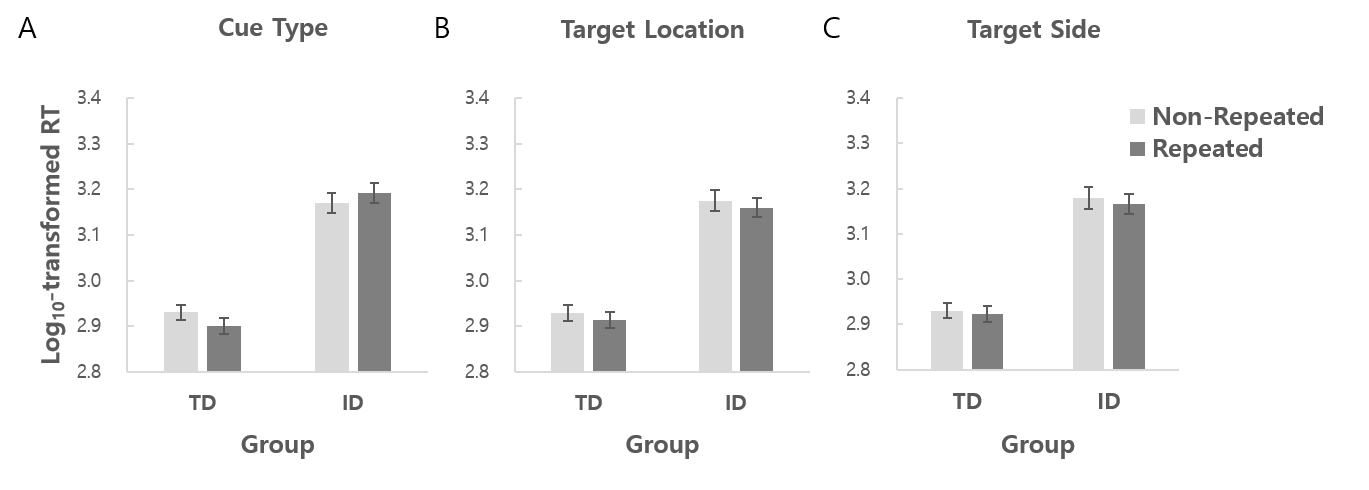


Figure . The repetition priming effect for different conditions. (A) Mean log_10_-transformed RT for cue type non-repeated vs. repeated trials in the TD and ID groups. (B) Mean log_10_-transformed RT for target location non-repeated vs. repeated trials in the TD and ID groups. (C) Mean log_10_-transformed RT for target side non-repeated vs. repeated trials in the TD and ID groups.

We also analyzed the effect of repetition priming of the *target location* (8 cube positions) and *target side* (left or right visual field), in the same manner with the log_10_-transformed RT data. The repetition of target location had a significant main effect [*F*(1, 38) = 8.36, *p =* .006, $\eta_{p}^{2}$ = .180] with faster RTs in target location-repeated trials (3.037) than in non-repeated trials (3.052), with no significant interaction between target location repetition and group [F(1, 38) = .003, *p =* .955, $\eta_{p}^{2}$ = .000] (Fig 4B). This indicates that both typically developing controls and individuals with ID showed the target location repetition priming effect in a similar way. Consistently, search efficiency was significantly better in target side-repeated trials (3.044) than in non-repeated trials (3.055) [*F*(1, 38) = 4.27, *p =* .046, $\eta_{p}^{2}$ = .101], with no significant interaction between target side repetition and group [*F*(1, 38) = .43, *p =* .52, $\eta_{p}^{2}$ = .011] (Fig 4C). Again, analyses based only on log_10_-transformed RT data reproduced the same pattern of results as in the IE-based analyses, that the function of the history-driven attentional mechanism that guides selective attention to the *previously attended location in space* is relatively preserved in ID.

**References**

Heitz, R. P. (2014). The speed-accuracy tradeoff: history, physiology, methodology, and behavior. *Frontiers in Neuroscience, 8*(150). doi:10.3389/fnins.2014.00150

Townsend, J. T., & Ashby, F. G. (1983). *Stochastic modelling of elementary psychological processes*. New York: Cambridge University Press.
