# Supplementary material for: Top-down, bottom-up, and history-driven processing of multisensory attentional cues in intellectual disability: An experimental study in virtual reality
Source: PLoS One. 2021 Dec 21;16(12):e0261298. doi: 10.1371/journal.pone.0261298 (PMC8691646; doi:10.1371/journal.pone.0261298)
Supplement: Attachment — Submitted filename: Response to Reviewers.docx [file pone.0261298.s002.docx]

**Academic Editor**

**Major points:**

*Point 1.* I'd like to thank the authors for providing their data on the OSF. However, I should note that I could not find a data-book (which provides the meta-data information) associated with the data. Please add a file to the OSF with the meta-data: describe what is presented in each column of the data-file, the valid values within the column and their meaning (e.g., what the cue-code number means in terms of the exact type of cue presented?). The matlab script does not include this information as well.

*Response.* We thank the editor for letting us know about a missing piece of information in the data on the OSF. We now have added meta-data files that describe the meaning of each column and value to the OSF.

*Point 2.* Please provide statistical justification for collapsing the data of the neutral cue conditions. Report an ANOVA and please provide the RT and accuracy means on a table for each cue type (this could also appear in a supplementary file).

*Response.* Since neutral cues were identical in terms of containing no information about the location of the upcoming target, we first checked the homogeneity in behavioral performance (IE) between neutral cue types by conducting a mixed ANOVA with neutral cue type (visual, auditory, tactile) as a within-subject factor and group as a between-subject factor. The results showed no significant main effect [*F*(2, 76) = 1.82, *P* = .17] or interaction [*F*(2, 76) = .28, *P* = .758] involving neutral cue type. Thus, search performance from neutral cue trials were collapsed in subsequent analyses. This information has been added in the manuscript (p. 14-15). We also have created a supplementary file, in which RT and accuracy means for each cue type are reported in a table (supplementary file p. 1).

*Point 3.* I did not follow the description of the feedback manipulation. It is mentioned that there were trials with and without feedback, but then the authors state that there were 4 feedback blocks for a total of 200 trials. What about the no-feedback blocks? Are there no-feedback blocks? Are the data from these blocks being analyzed? In the end of the results section it is mentioned that there were some evaluation of the effect of feedback. It is not clear how this was done.

*Response.* The experiment consisted of four feedback-type blocks, namely no-feedback, visual feedback, auditory feedback, and tactile feedback, with the order of blocks determined randomly for each participant. Each feedback block consisted of 50 trials, in which all types of cues were randomly intermixed. The data from all four blocks were analyzed to observe the effect of cue type manipulations. In the end of the results section, we additionally analyzed whether the four feedback type (no-feedback, visual feedback, auditory feedback, and tactile feedback) influenced the overall search performance by entering the IE data into a mixed ANOVA with feedback block (visual, auditory, tactile, and no-feedback) as a within-subject factor and group as a between-subject factor. The results showed no significant main effect [*F*(3, 114) = .64, *P* = .59] or interaction [*F*(3, 114) = .67, *P* = .57] involving feedback block, indicating that getting different types of feedback on the accuracy of the response did not influence the overall search performance. We now have added clearer description of the feedback block manipulations and analysis in the manuscript (p. 15).

*Point 4.* Overall, I’d caution the authors against manipulating so many variables in a single experiment in the future. The number of trials per design cell seems rather in the low end. I want the authors to report exactly how many trials per design cell they have. Overall, they should not report analyses for which they would have fewer than 20 responses per design cell, as this is likely to be unreliable. Besides that, the authors mention 10 types of cues, but I counted 12 types of cues. Are they already assuming that the neutral cues form a single condition in the break-out of the cues?

*Response.* We agree with the editor’s comment that manipulating many variables in a single experiment is not desirable, and will keep that in mind in the future. Regarding the number of trials per design cell, the *smallest* design cell (e.g., visual exogenous motion cue) contained 20 repetitions of trials. We acknowledge that it is rather in the low end, but considering participants’ fatigue from taking part in a long VR experiment and particularly slower overall response times of the ID group, we had to compromise at 20 repetitions for the smallest design cell, which is generally considered to be reliable. Regarding neutral cues, the three types (visual, auditory, tactile) of neutral cues were identical in terms of containing no information about the location of the upcoming target. Thus, we assumed that there would be no significant difference in search performance from the trials in which visual, auditory, or tactile neutral cues were presented, and it was confirmed in a mixed ANOVA that revealed no significant main effect [*F*(2, 76) = 1.82, *P* = .17] or interaction [*F*(2, 76) = .28, *P* = .758] involving neutral cue type. We therefore collapsed the neutral trials into a single condition and used it as a baseline performance for each participant.

*Point 5.* The use of inverse efficiency scores has been cautioned against in the literature (see for example Bruyer and Brysbaert, 2011) unless some conditions are met: relative high accuracy (which was the case here) but also absence of speed-accuracy tradeoffs. Can you please indicate whether the RTs and PE go in the same direction by presenting their correlation? Overall, it would be reassuring to see that analyses based only on RTs reproduce the same pattern of results. These supplementary analysis only on RT could be reported in supplementary file. In fact, given the much slower RTs of the ID group, it might be more appropriate to analyze logRTs. This would make sure that the interaction between group and cue is not due to the proportional slowing of the RTs in the ID group (similarly to the concerns raised in the aging literature). This is important, since this would make sure the interaction is not simply an artifact of the perceptual/motor-speed difference between groups, but indeed reflect an effect on attention guidance. If the authors present these analysis in the supplementary file, they should nevertheless indicate in the main text if the interaction was still significant in this analysis.

*Response.* Following the editor’s suggestion, we correlated each cue condition’s mean RT and mean accuracy rate to examine the possible speed-accuracy tradeoff. For the TD group, there was a significant *negative* correlation between RT and accuracy rate (*r* = -.80, *P* = .002), indicating that the conditions with higher mean accuracy rates also tended to have faster mean RTs. This is the *opposite* of the definition of speed-accuracy trade-off. For the ID group, the correlation between RT and accuracy rate was not significant (*r* = .45, *P* = .143), showing *no sign* of the speed-accuracy tradeoff. This information has been added to the manuscript (p. 17-18) and the supplementary file (p. 2-3). Regarding analyses based only on logRTs, all the major effects we observed with the IE data were replicated with the log_10_-transformed RT data. Detailed results have been reported in the supplementary file (p. 3-8) and also indicated in the main manuscript (p. 18).

**Minor points:**

1. l.320-324: The separate ANOVAs do not show that the effect is smaller in one condition than the other. They show that there is a main effect of cue in both groups, which renders probable that the interaction is due to the smaller effect in the ID group.

*Response.* We agree with the editor that separate ANOVAs do not actually prove that the effect is smaller in one condition than the other. We now have added the result of an independent samples t-test to support the statement that the difference in mean IE between neutral cues versus informative cues was smaller in the ID group (233 ms) than in the TD group (435 ms) [*t*(38) = 3.48, *P* = .001] in the manuscript (p. 20).

2. l.345-346: The results of the separate ANOVAs are not being reported. Please report the actual statistics associated with each of them, instead of simply reporting it verbally. Besides that, it is not proper to only report the p-value for a comparison of cue types in the ID group. Report the full statistical analysis.

*Response.* We now have reported full statistics associated with each test throughout the results section in the manuscript.

3. I take the point from Reviewer 1 that the arrow cues can be considered as a hybrid, hence the conclusion that endogenous cues in the visual modality is preserved should be made with caution and the relevant literature on the status of arrow cues should be reviewed.

*Response.* Following the editor and reviewer’s suggestion, we now have addressed the possibility that the arrow cues may engage both voluntary and reflexive attention, and reviewed the relevant literature on the status of arrow cues in the discussion section of the manuscript (p. 27-28). Please also refer to our response to Reviewer 1’s Point 2 below.

4. Overall, please report the degrees of freedom for all statistical tests, including the t-tests!

*Response.* We now have reported the degrees of freedom for all statistical tests in the manuscript.

**Reviewer 1**

**Major points:**
*Point 1.* It is unclear to what degree the less prominent orienting on the basis of endogenous cues in ID is due to a deficit in top-down attention or to an impoverished ability to apply task instruction more generally. As I understand it, the practice session ensures the ability to correctly report the target within the timeout limit, not to interpret the meaning of the cues. Particularly given the diversity of cue conditions, there is a lot of information for participants to track in this paradigm, and participants with ID may have simply been more likely to struggle understanding the rules concerning the meaning of the cues at all (hence the near-zero cuing effects in some cases) and/or lose track of these rules on a subset of trials. Were this the case, the data would seem hardly surprising and probably just a reflection of impaired cognition.

*Response.* In the practice session, at least two experienced experimenters constantly monitored not only the participant’s behavioral performance (accuracy) but also the understanding of each cue type on each trial. We constantly checked if the participant understood the meaning of each cue type by observing the head and eye movement of the participant via the screen that showed the current VR visual field of the participant: Participants who understood the meaning of the cue oriented towards the side (left or right) indicated by the cue immediately after the presentation of the cue. When the participant showed no sign of understanding the cue information, experimenters immediately intervened and explained the rules again. Importantly, instructions and practice blocks were *repeated* until all experimenters agreed on the participant’s successful understanding of the meaning of each cue type and a high level of accuracy rate (over 90%). As the reviewer’s comment, it took much longer for the ID group to successfully learn the meaning of cues, sometimes repeating the practice session multiple times. Overall, the ID group spent approximately twice as much time (20-30 minutes) on instructions and practice blocks as compared to the TD group. Since the main experimental task was conducted only if all experimenters agreed upon the participant’s successful understanding of the task and the meaning of each cue type, it is hard to conclude that the observed differences between groups resulted entirely from the impaired ability to apply task rules in general. This has been more clarified in the manuscript (p. 15-16).

*Point 2.* The endogenous visual cues are quite different from the other endogenous cues with respect to their intuitiveness. In fact, there is evidence that arrow cues can to some degree function as exogenous cues (and produce cuing effects even when non-predictive), likely due to their highly overlearned meaning (which contrasts with the other endogenous cues in this experiment). As such, the intact cuing effects for the "endogenous" visual cues in ID may simply reflect a substantially reduced role for task instruction producing those cuing effects (and thus be consistent with the possibility outlined in point 1). I would also strongly caution against drawing conclusions concerning a sparing of endogenous attention in the visual modality: it may well have nothing to do with the modality and everything to do with the specific choice of cues.

*Response.* We agree with the reviewer that the strength of directional associations was not matched between central arrow cues and other endogenous cues, because the meaning of an arrow is learned repeatedly throughout life. However, it was practically impossible to identify symbolic cues in auditory or tactile modality that have similar intuitiveness as visual ones, since most people without sensory disabilities tend to rely heavily on the *visual* symbolic system. For instance, we first thought of using verbal symbols, such as the words ‘left’ or ‘right’ in visual and auditory modalities, but there were no comparable tactile verbal symbols. Moreover, clinicians who screened the ID participants informed us that people with ID often confuse the left and right side indicated by verbal directions, which suggests the possibility that the strength of associations between directional words and the meaning of them is not comparable between ID and TD groups. This would be particularly problematic, because the purpose of our manipulation was to measure the relative strength of *attentional* orienting, not the ability to interpret the meaning of words. Thus, we used the arrow direction that both ID and TD participants could intuitively utilize to orient attention to the indicated direction. Auditory and tactile endogenous cues were then designed to make them as similar as possible to the arrow cue, utilizing the direction of the sound source movement or the vibration source movement. We also let participants imagine an arrow flying in a specific direction, making a sound or brushing the hands, when explaining about the auditory and tactile endogenous cues.

Previous studies showed that central symbolic cues that are highly overlearned or have social importance, such as arrows (Bayliss, Di Pellegrino, & Tipper, 2005; Marotta, Lupiáñez, Martella, & Casagrande, 2012), gaze direction (Driver et al., 1999), finger pointing (Langton & Bruce, 2000), and words indicating a spatial direction (Hommel, Pratt, Colzato, & Godijn, 2001) can orient attention in a reflexive manner even if they are not predictive about the target location. Ristic and Kingstone (2006) also argued that predictive arrow cues engage *both* volitional and reflexive attention, with these effects combining in an interactive manner. Therefore, some researchers suggested that central cues should be truly symbolic, such as a color cue arbitrarily associated with different directions, in order to examine pure voluntary attentional orienting (Chica, Martín-Arévalo, Botta, & Lupiáñez, 2014; Dodd & Wilson, 2009). Unfortunately, we could not utilize such pure arbitrarily-learned symbols as endogenous cues in this study, considering the significantly impaired learning abilities in ID participants as compared to TD participants. As stated above, what we wanted to examine was the strength of attentional orienting, not the ability to learn and interpret the meaning of arbitrary symbols. We now have added relevant literature review on the status of arrow cues, caution against the conclusions regarding a sparing of visual top-down attention, and the directions for future studies with pure arbitrary endogenous cues in the discussion section (p. 27-28).

*Point 3.* We do not know whether the "exogenous" cues were really effective as exogenous cues, since there are no invalid trials and the cues are 100% informative. These are really just more intuitive endogenous cues that may or may not have an exogenous component. As with the prior points, this really muddles the interpretation.

*Response.* We included ‘neutral’ cues, instead of ‘invalid’ cues, since including invalid trials and manipulating the probability between valid and invalid trials (e.g., 80%/20%) should involve *statistical learning abilities*, which are reported to be significantly different between TD and ID groups (Saffran, 2018), and therefore would confound the results obtained from the two groups. Also, the inclusion of invalid trials alone cannot actually guarantee that the effects of cues are purely involuntary, since participants might still show a behavioral bias to orient towards the cued side even when the predicted validity of the cue is less than 100%. In the original spatial cueing paradigm (Posner, 1980), there were three types of trials, valid, neutral, and invalid, and the performance in the neutral trials was used as a baseline to observe the cueing effect of both central (endogenous) and peripheral (exogenous) cues. Thus, we used ‘neutral cues’ that provided no information about the upcoming location of the target as a baseline, and calculated the cue effect index by subtracting the IE value of each cue type from that of the neutral cue for each participant. The rationale for using neutral cues has been more elaborated in the manuscript (p. 14).

*Point 4.* Why are the selection history effects with respect to the cue not broken down by cue type (modality and endogenous vs. exogenous)? Especially in the case of endogenous auditory and tactile cues, this really is not a fair comparison since those cues did not really show much of any cuing effect in ID (and more generally, if the ID participants struggled to use those cues, there is really not much in the way of selection that would repeat). And what about whether the direction of the cue repeated or changed (and not just the type of cue)? There is quite a bit going on here that could really change the interpretation. As things currently stand, I would strongly hesitate to conclude that selection history per se is fundamentally altered in ID without a more careful look at this data.

*Response.* The repetition priming effect was not broken down by each cue type, in order to secure the enough number of trials for repeated vs. non-repeated conditions. Since there were many cue types in our study, the number of immediately repeated trials broken down by each cue type would be too small for reliable analysis. Regarding the absence of cueing effect in endogenous auditory and tactile cues, the examination of the repetition priming effect was independent from the cueing effect analyses. We divided trials into repeated and non-repeated subsets depending on whether the cue type on the current trial (n) is the same as that on the previous trial (n-1) or not, and observed whether there is improvement in *IE (not cueing effect)* in repeated trials as compared to non-repeated trials. Thus, regardless of whether a cue had a significant cueing effect or not (as compared to the neutral cue trials), what we examined was whether the consecutive experience of the same cue type changed the search performance or not. Regarding the repetition priming effect with respect to the direction of the cue, we already analyzed it by examining the repetition priming effect of *target side*. In our study, cues were 100% valid and therefore the cued side and the target side coincided. The results indicated that both TD and ID groups showed marginally better search performance in target side-repeated trials than in non-repeated trials (p. 23-24).

*Point 5.* Given that this was a study on ID, I was a little surprised that the authors designed the task such that the target digit changed unpredictably trial-to-trial and required tracking. This demand may have made it more difficult for the ID participants to maintain the rules about how to interpret the endogenous cues. More broadly, this manipulation could have been better justified.

*Response.* The search target varied across trials and was cued at the start of each trial, following the typical visual search paradigm that has often been used in attention literature (Carlisle, Arita, Pardo, & Woodman, 2011; Woodman, Luck, & Schall, 2007). The potential target was confined to a one-digit number (0~9), which we assumed not to be such a great burden on the working memory capacity. This assumption was confirmed by very high mean accuracy rate (98.2%) even in the ID group, indicating that they could successfully track the changing search target on each trial. In addition, the search target cued at the start of each trial had a function of attracting the participant’s gaze towards the center of the 3-D background, such that participants start their search from the center, rather than shifted towards the left or right side.

*Point 6.* For a patient study, the sample size seemed surprisingly small. There is no justification given for powering the study to detect a "medium" effect size, nor does the power analysis take into account the large number of conditions and the many analyses conducted. Also, for such a modest sample size, it is surprising that the samples were not more closely matched (esp. with respect to sex) -- given that the control sample is university students this should not be difficult to better match. With the low sample size and number of conditions, there really is not sufficient power to know whether an imbalance in participant characteristics is influencing the results at all, particularly as they might interact with ID.

*Response.* In this study, the main research question was to examine how the processing of attentional cues differ between people with intellectual disability and typically-developing controls. Thus, the main analysis of interest was the *interaction between group* *(ID, TD) and cue type (sense, origin)*. We therefore determined the number of participants a priori by a power analysis (G*power version 3.1.9.2), using parameters corresponding to the main analysis of interest, the three-way interaction between group (ID, TD), sense (visual, auditory, tactile), and origin (endogenous, exogenous), with .85 power to find a medium-size effect ($\eta_{p}^{2}$ = .06) in a mixed ANOVA within-between interaction test (number of groups = 2, number of measurements = 6, alpha = .05). The medium-size effect was used in the power analysis based on the previous studies that compared the effect of attentional cues on the behavioral performance of a developmental disability (ID or ASD) group with that of a typically-developing group (Amso, Haas, Tenenbaum, Markant, & Sheinkopf, 2014; Chiviacowsky, Wulf, & Avila, 2013; Greenaway & Plaisted, 2005; Merrill, Conners, Yang, & Weathington, 2014). In these studies, the reported effect sizes for the interaction between group and cue type were all *medium to large* ($\eta_{p}^{2}$ = .06 ~ .24). Thus, we conservatively used a medium-size effect ($\eta_{p}^{2}$ = .06) to perform a power analysis, which corresponded to Cohen (2013)’s definition of the conventional medium effect size at d = .5. Consistent with the previous studies, we also observed large effect sizes ($\eta_{p}^{2}$= .213 ~ .358) of the interaction between group and cue type. We now have added detailed information and justification of the power analysis in the manuscript (p. 8). Regarding the homogeneity of the ID and TD groups, we additionally compared gender and handedness between groups using Pearson’s chi-squared tests. The results confirmed that the ID and TD groups were not significantly different in terms of gender (χ^2^= 1.758, df =1, *P* = .185) and handedness (χ^2^= 2.090, df =2, *P* = .352) compositions. This is now added in the manuscript (p. 9).

*Point 7.* Accuracy was very high in this task. It was therefore quite unexpected that the authors would focus their analyses on inverse efficiency (IE) rather than RT, since accuracy does not seem to be an informative measure in this situation and is likely adding noise to the data. This contrasts markedly from the kind of situations for which IE was developed, where both accuracy and RT are clearly influenced.

*Response.* The use of inverse efficiency scores is deemed appropriate when several conditions are met: Relative high accuracy, which was the case in our experiment, and also the absence of the speed-accuracy tradeoff (Bruyer & Brysbaert, 2011). In order to examine whether there was the speed-accuracy tradeoff or not, we correlated each cue condition’s mean RT and accuracy rate. For the TD group, there was a significant negative correlation between RT and accuracy rate (*r* = -.80, *P* = .002), indicating that the conditions with higher mean accuracy rates also tended to have faster mean RTs. This is the opposite of the definition of speed-accuracy trade-off. For the ID group, the correlation between RT and accuracy rate was not significant (*r* = .45, *P* = .143), showing no sign of the speed-accuracy tradeoff. Moreover, preliminary analyses on RT and accuracy rate revealed that there were significant effects involving cue type in *both* RT and accuracy rate data. Thus, inverse efficiency (IE) values were calculated and analyzed for each participant/cue condition to combine the effects of RT and accuracy in all subsequent analyses. We also conducted analyses based only on log_10_-transformed RTs, and reproduced the same pattern of results. This is now indicated in the manuscript (p. 17-18) and detailed results are reported in the supplementary file (p. 1-3).

*Point 8.* Why were eye and/or head movements not analyzed and reported? As a study on attentional cuing, these measures could be more informative than RT/IE.

*Response.* We agree with the reviewer that analyzing eye/head movements, in addition to RT/Accuracy, would be informative in examining overt attentional characteristics of participants. In this experiment, Oculus Rift S, which has no built-in eye-tracker, was used as a VR HMD. Thus, we could only observe large eye/head movements via the screen that showed the VR visual field of the participant, but could not record precise fixations and saccades. We are currently using VR HMDs that have a built-in eye tracker (HTC Vive Pro Eye) or add-on eye-tracker (Pupil labs) in the lab for future studies.

*Point 9.* The somewhat dramatic difference in mean RT between groups could be inducing some sort of ceiling effect in the ID group. More should be done to try to rule this out with the data.

*Response.* To rule out the possibility that the observed interaction between group and cue type was due to the proportional slowing of the RTs in the ID group, we conducted additional analyses with log_10_-transformed RTs, and replicated all the major effects we observed with the IE data, including the significant interaction between group and cue type. This is now indicated in the manuscript (p. 18), and detailed results are reported in the supplementary file (p. 3-8).

**Minor points:**
1. I would note that IE should not be expressed in ms. It is really an arbitrary unit that is no more linked to ms than it is to proportion correct.

*Response.* Milisecond was used as a unit for IE, since IE is calculated by dividing the average RT (ms) by accuracy rate (no unit) of that condition. The IE is identical to the average RT when the accuracy rate is 1, and it linearly increases as the accuracy rate decreases. In order to make the comparisons between conditions more intuitive, we used ms as a unit for IE, rather than having no unit at all. Many other previous studies also used ms as a unit for IE (Bruyer & Brysbaert, 2011; Kennett, Eimer, Spence, & Driver, 2001; Kuefner, Cassia, Vescovo, & Picozzi, 2010).

2. Some of the ANOVAs have overlap in the effects reported, with some identified effects to some degree restating each other (e.g., multiple main effects of group). I am not sure that this is inherently a problem, although the authors might be a little more careful in how they emphasize/contextualize different effects when this happens (make sure the reader understands the redundancy and that this does not constitute converging evidence).

*Response.* Following the reviewer’s suggestion, we have made it clearer that our main analysis of interest is the *interaction* between group and cue type. The main effects of group were reported only to fully address all statistical results, and we did not emphasize the multiple group effects as constituting converging evidence.

3. "highly unnatural conditions" (line 462). It is not clear to me to what degree the present study really marks an improvement in this respect. Yes, it is in a 3-D rendered environment, but the task is still quite a bit artificial (in a variety of ways).

*Response.* The majority of previous studies investigated how the aspects of visual stimuli presented on a 2-D screen guide covert attentional selection, while observers’ *head and eye movements were strictly restricted* and responses were made by pressing *arbitrarily associated input devices* (e.g., keyboard, buttons). Much fewer studies investigated the attentional process within other sensory modalities in natural conditions where participants are allowed to freely *move their eyes and head* in a *3-D environment* and *responses are made via intuitive body movements* such as reaching, touching, or grasping the object of interest itself. Using VR technology, we systematically delivered visual, auditory, and tactile stimuli to observers in a 3-D environment, in which they could freely move their eyes and head to search for an object, and made responses by reaching and touching the found target with their own hands (tracked by controllers). We believe this is a huge improvement in terms of naturality of the experimental task, since in everyday life, humans search for a specific object (e.g., a car key) by sampling the 3D environment with head and eye movements, and naturally move body parts to acquire the found target object (e.g., reaching out and grabbing the car key with arm and hand movements).

**Reviewer 2**

**Major points:**

*Point 1*: The work reported is important and well grounded (and authors made that clear). Writing is very good in terms of coherence and clarity (some proofreading may be needed just to grasp some minor issues, sometimes the use of articles seemed to be missing). My only concern, easily solved through re-writing, is the lack of clear research questions and hypotheses, explicitly matched with the data analytic plan (which could be presented in a more systematic way, rather than “as we read”).

*Response.* We thank the reviewer for recognizing the merits of our manuscript. Following the reviewer’s suggestion, we now have clearly described the research questions, hypotheses, and explicitly matched data analytic plan in the main manuscript. Please refer to our responses to specific comments below for details.

**Minor points:**
1. In the introduction I felt the need of some more (not too much) information about the intellectual disability (ID) as a neurodevelopmental disorder.

*Response.* Neurodevelopmental disorders indicate neurologically based conditions that appear early in childhood, typically before school entry. In DSM‑5, neurodevelopmental disorders (NDDs) are defined as a group of conditions with onset in the developmental period, inducing deficits that produce impairments of functioning. NDDs include ID, ASD, ADHD, Communication disorders, specific learning disorders, and neurodevelopmental motor disorders. We have added the phrase “ID is a neurodevelopmental disorder that appears early in childhood…” in the manuscript (p. 3).

2. On p. 4, starting on line 85, authors are introducing a third mechanism, but this only becomes clear in the middle of the paragraph. In the beginning, readers are kind of lost trying to get why that info is relevant. Making it clear up front may help understanding.

*Response.* We now have clearly introduced the third attentional mechanism up front in the beginning of the paragraph (p. 4).

3. On p. 5 the starting of the paragraph with “another issue” seems also a bit disconnected (something like “by the way”). It’s a minor aspect but I’d like to challenge authors to try a better transition from the presentation of the mechanisms to the modality/artificial conditions issue.

*Response.* Following the reviewer’s suggestion, we have revised the beginning phrase of the paragraph for better transition (p. 5).

4. In the last paragraph of p. 6, when the study is presented, authors refer to “issues”. I believe it would be better to present research questions and hypotheses (though a general hypothesis is presented for issue #1, it was not for issue #2).

*Response.* We now have revised the paragraph such that our research questions and hypotheses are clearly presented (p. 6-7).

5. On top of p. 7, authors say “with two different feature dimensions in each modality (e.g., color and motion features for visual modality)”. The purposes of these two dimensions (for visual and the other modalities) is not clear.

*Response.* The paragraph has been revised such that the focus is on the two main research questions and hypotheses (p. 6-7). Additional exploratory analyses, including comparisons between different feature dimensions and different feedback blocks, are described later in the methods section to focus on the main research questions and avoid confusion (p. 13, 15).

6. Next authors refer to “compare behavioral performance across conditions”. It would be helpful to clarify exactly which kind of behavioral performance. This may also help to understand what is meant by “efficiency”.

*Response.* We now have changed the sentence as “compare the behavioral performance (response time and accuracy rate to find the search target)…” to clarify what exactly was measured (p. 7).

7. I’d like to praise authors for including power analyses. However, as several analyses were done, I was not sure about what was used (mainly in terms of the within subject factor). Also, authors refer to the interactions, but what about the main effects? Authors could additionally include a citation to support the decision of the effect size used as reference.

*Response.* In this experiment, the main research question was to examine how the processing of attentional cues differ between people with intellectual disability and typically-developing controls. Thus, the main analysis of interest was the *interaction between group* *(ID, TD) and cue type (sense, origin)*. Thus a power analysis (G*power version 3.1.9.2) was conducted using parameters corresponding to the main analysis of interest, the interaction between group (ID, TD), sense (visual, auditory, tactile), and origin (endogenous, exogenous), with .85 power to find a medium-size effect ($\eta_{p}^{2}$ = .06) in a mixed ANOVA within-between interaction test (number of groups = 2, number of measurements = 6, alpha = .05). The medium-size effect was used in the power analysis based on the previous studies that compared the effect of attentional cues on the behavioral performance of a developmental disability (ID, ASD) group with that of a typically-developing group (Amso et al., 2014; Chiviacowsky et al., 2013; Greenaway & Plaisted, 2005; Merrill et al., 2014). In these studies, the main analysis of interest was the interaction between group and cue type just as in our study, and the reported effect sizes were all medium to large ($\eta_{p}^{2}$ = .06 ~ .24). Thus, we conservatively used a medium-size effect ($\eta_{p}^{2}$ = .06) to perform a power analysis, which corresponded to Cohen (2013)’s definition of the conventional medium effect size at d= .5. Consistent with the previous studies, we also observed large effect sizes ($\eta_{p}^{2}$= .213 ~ .358) of the interaction involving group and cue type. We now have added detailed information and justification of the power analysis in the manuscript (p. 8).

8. Authors used inferential statistics to compare age between groups. Any reason for not comparing at least gender and handedness? Looking at the frequencies, they seem similar, but a statistical test would give more confidence on the groups equivalence.

*Response.* Following the reviewer’s suggestion, we compared gender and handedness between groups using Pearson’s chi-squared tests. The results confirmed that the ID and TD groups were not significantly different in terms of gender (χ^2^= 1.758, df =1, p = .185) and handedness (χ^2^= 2.090, df =2, p = .352) compositions. This is now added in the manuscript (p. 9).

9. On p. 9, line 175 it is said that participants with clinical history of other neurodevelopmental problems (e.g., Down syndrome) were excluded. But looking at the table, we can see those with ASD were kept. Any reason for this double criterium?

*Response.* A recent review reported that the comorbidity rates of ID and other mental disorders are very high, between 30% and 50% (Einfeld, Ellis, & Emerson, 2011). In particular, ID and ASD covary at very high rates, and the covarying rates are even more increased after changes in diagnostic criteria (Matson & Shoemaker, 2009). For instance, LaMalfa, Lassi, Bertelli, Salvini, and Placidi (2004) reported that 40% of persons with ID have an ASD, while 70% of persons with ASD have ID. Bryson, Bradley, Thompson, and Wainwright (2008) found that 28% of individuals with ID also evinced autism. Considering the high comorbidity rates of ID, we did not exclude ASD-comorbid participants to secure the enough number of participants, provided that they fitted our inclusion criteria: *a diagnosis of mild to moderate intellectual disability* with recognized ability of basic verbal communication. Instead, we excluded people who showed any significant perceptual (e.g., visual, auditory, tactile) or motor deficits in clinical observations or in the Adolescent/Adult Sensory Profile, since it could directly influence the behavioral performance in our experimental task. We also excluded people who had a clinical history of other neurodevelopmental genetic disorders (e.g., Down syndrome, Williams syndrome), which are known to have distinct characteristic symptoms and features. We now have described the inclusion and exclusion criteria in more detail in the main manuscript (p. 9-10).

10. Concerning the other reason for excluding “any other behavioral problems”, not clear what this means exactly (maybe adding an example) and how was done. An indication of how many participants were excluded would be helpful as well as making it clear that the 40 were after exclusions.

*Response.* People with intellectual disabilities frequently have some forms of behavioral disorders that may manifest as stereotyped behaviors, difficult or disruptive behavior, aggressive behaviors toward the self, other people, or property (Lowe et al., 2007; Smith & Matson, 2010). In order to ensure safety and completion of participation, people who had a history of behavioral problems (e.g., stereotyped behaviors, difficult, disruptive, or aggressive behavior) were *prescreened* by independent clinicians at the vocational training center for developmental disabilities. Thus, 40 is the final sample size, after exclusions. This information is now added to the manuscript (p. 10).

11. As far as I understood, in the experimental task, authors manipulated cue and feedback, but also cue feature dimensions. Maybe that could be said up front before presenting the different manipulations. In particular, the feedback part, I just got the whole point of it after reading the whole paragraph.

*Response.* We have now clearly described the manipulation of cue feature dimensions and feedback at the beginning of each paragraph (p. 13, 15).

12. On a related vein, though it may be there implicitly, I think authors should establish a clear link between the theory/hypotheses and the conditions. For example, the endogenous/exogenous and modality parts are quite obvious, but the role of the feedback and the dimensions (color/motion, etc.) was not.

*Response.* The purposes of the manipulation in feature dimensions and feedback are now more clearly stated in the manuscript (p. 13, 15)

13. The description of the experimental task is clear. I did however miss an explicit indication about the measures extracted from the task (I believe this point relates with the comments above on behavioral performance and efficiency). This may also help to understand the exact meaning of “inversed efficiency”. The formula is clear but the meaning not so.

*Response.* We have explicitly indicated that the response time and accuracy of the search response was measured on each trial in the manuscript (p. 12), and added a sentence that explains the meaning of inverse efficiency (“higher IE values generally indicate lower search efficiency (longer RTs and/or lower accuracy”) (p. 16).

14. Still about the analysis, authors mentioned a mixed ANOVA but just present the between subject factor. A clear presentation of the factors seems needed.

*Response.* We now have added the phrase “…mixed ANOVAs with group as a between-subject factor and different cue type (origin, sense, or repetition) as within-subject factors” in the manuscript (p. 17).

15. Perhaps is a question of writing, but I didn’t get why main effects and interactions were followed with separate analyses (which may increase Type error I rate) instead of pairwise comparisons or post-hocs for main effects and simple effects analyses for interactions, within the ANOVA (and not with additional tests). The result might be the same, but the approach is much more parsimonious and reduces Type error I. An additional advantage is that, in the interactions, authors will be able to compare not only condition differences separately by group but also group differences separately by conditions (this latter part was not examined, and may help to understand findings).

*Response.* We first performed mixed ANOVAs with group as a between-subject factor and cue type as within-subject factors, and only if there were significant interactions involving group, separate repeated-measures ANOVAs for the two groups were conducted. Here, the separate repeated-measures ANOVAs for the two groups were conducted *as post-hoc comparisons* to closely examine the observed interactions involving group. It is conceptually the same as conducting paired t-tests only when a significant main effect is observed to reduce the Type I error.

16. Despite that section explaining the analyses before the results, when we go through that section, we realize that other analyses were conducted, with varying within subject factors, and sometimes with unclear purposes. I understand this might be the “response” for the comment #14 above, but it is a bit confusing and hard to follow. The analyses should be all explained before the results section.

*Response.* By revising the methods section as stated in our responses to the reviewer’s previous comments above, we believe all analyses are now introduced before the results section.

17. If authors used the inversed efficiency score, which combines accuracy and RT, why it is said on p. 15 lines 301-302 that only RT for correct trials were analyzed? This would mean that for compute the inversed efficiency score, the accuracy one would be a constant.

*Response.* The IE is calculated by dividing the mean RT of each condition by the accuracy rate of that condition for each participant. “only RT for correct trials were analyzed” means that when calculating the mean RT of each condition, only the RTs on correct trials were averaged. Excluding RTs on incorrect trials when calculating the condition mean is a commonly used standard procedure in RT analyses.

18. Through the results section I believe P should be changed to p (lowercase).

*Response.* Following the PLOS ONE guideline, uppercase P was used when reporting p-values.

19. Overall, the results section is not aligned with the present study description and is a bit hard to follow, mainly because readers don’t have a clear idea about what research question is each analysis responding to, and also because authors mixed explanation for the analyses with results and discussion statements.

*Response.* We now have revised the results section, such that the correspondence between each research question, analysis, and the results are more clearly expressed.

20. On p. 18 lines 374 authors mention “six feature dimensions, namely color, motion, verbal, nonverbal, high-frequency, and low-frequency”. This is mentioned briefly in the method but the purpose of comparing this was not clear. This happens with several other analyses (e.g., target side/location, feedback, etc.)

*Response.* Sentences that explain the purpose of conducting secondary exploratory analyses (feedback, feature dimensions, etc.) have been added throughout the manuscript.

21. My main concern with this manuscript is that there is not clear match between the research questions, how each one was methodologically addressed, what are the hypotheses for each one, and which analyses were used to test each and every one of them. Only in the results section, do we realize there are some “new” specific research goals, not fully introduced in the lit review.

*Response.* By revising the methods and results section in response to the reviewer’s previous comments, we believe that the correspondence between our main research questions, hypotheses, and analyses have become clearer.

22. This match between RQ/hypotheses/analyses will also help to organize the discussion. As it is (mainly the first/second paragraphs) is clear and insightful, but I believe it could be expanded. This could be done by addressing the results of the analyses in full as well as by going beyond a simple summary of the findings and interpreting them. I’d also like to read something more about the implications of the findings for theory. The introduction is strongly framed to claim on the novelty of the study and on the overcoming gaps in the literature. But then the discussion is more “modest” and does not establish that link back to theory. Some indications for future research would also be useful.

*Response.* Following the reviewer’s suggestions, we now have expanded the discussion section a lot, addressing the interpretations of our results and possible alternative hypotheses, review on related works and theories, and the direction for future studies (p. 26-29).

**Reviewer 3**

**Minor points:**
1. I appreciate that the authors provide information about the severity of intellectual disability in their sample overall, however given substantial heterogeneity in this population additional descriptive information would be helpful in integrating their work with the field. Are IQ scores available? Also scores on any standardized measures of adaptive functioning? Is there any additional standardization of diagnosis or any specific medical or other comorbidity rule-outs for participants in this sample?

*Response.* Until the DSM-4, the ID severity levels were based only on the IQ scores. For instance, mild ID had an approximate IQ range of 50~69, and moderate ID had an approximate IQ range of 36~49. However, the DSM-V abandoned specific IQ cutoffs as a diagnostic criterion, and placed more emphasis on the impairments in conceptual, social, and practical life skill domains. For instance, the mild ID is described as an individual who “can live independently with minimum levels of support”, whereas the moderate ID is described as “independent living may be achieved with moderate levels of support, such as those available in group homes” (APA, 2013). Following the DSM-5 criteria, each participant’s diagnosis and severity level of ID was determined by independent clinicians based on comprehensive evaluation of the scores on standardized tests, including IQ test (K-WAIS), Peabody Picture Vocabulary Test (K-PPVT), Bender Gestalt Test (BGT), Individual Basic Learning Skills Test (K-IBLST), Hand Function Test, Work Sample Test, and also clinical interviews and observations. Specific scores were available only to the independent clinicians, and they prescreened candidates according to our inclusion and exclusion criteria. Detailed explanation about the diagnosis and inclusion/exclusion criteria has now been added in the manuscript (p. 9-10).

2. In several instances authors seem to lump ID with ADHD and ASD (e.g., in justifying why top-down attentional problems may be present). While these are all developmental disabilities and ID may occur in higher rates in ADHD and ASD, they are also fundamentally different populations. Indeed many individuals with ASD and ADHD have normal or above average IQ and adaptive skills. This should be clarified throughout the manuscript. Additional discussion related to whether we would expect the same mechanisms of attentional problems in these populations or might expect different attentional patterns (e.g., more general deficits in one group versus more specific in another diagnostic group) will add important nuance to the current discussion.

*Response.* We agree with the reviewer’s comment that ID, ASD, and ADHD are fundamentally different populations and it would be important to discuss about whether they share the same mechanisms of attentional problems or not. It was not our intention to lump ID with ADHD and ASD, and we now have made it clear throughout the manuscript. Our speculations and relevant literature about different attentional patterns in those three conditions are also added in the discussion section (p. 26-27).

3. The authors find an interesting pattern in which the history-driven attentional guidance may actually operate in a counter-productive (and counterintuitive) manner in those with ID. Is there any prior research or theory that might help explain this finding? Some additional discussion of how the patterns map onto prior work in ID, known differentiation of networks supporting history-driven attentional guidance, or other possible theoretical explanations for this finding will help readers understand whether this pattern makes sense or is more likely to be a fluke of current data.

*Response.* History-driven attentional guidance is closely intertwined with implicit learning and memory functions. In the current study, the repetition priming effects for the specific target location and target side were observed in both ID and TD groups, suggesting that the function of the history-driven attentional mechanism that guides attention to the previously attended *location in space* was still preserved in ID. This result is in line with the assertion that implicit learning is an evolutionarily primitive form of learning and should not vary with age and general intelligence (Reber, 1992). Consistently, previous studies that involved implicit learning of *visual-spatial* information, such as contextual cueing (Merrill et al., 2014) or repetition priming of visual perception (Wyatt & Conners, 1998), reported comparable facilitation effects in ID and TD individuals. On the other hand, when repetition priming for complex *verbal* material was investigated, a contradictory pattern of results emerged, with some reporting comparable priming effects for ID and TD groups (Vicari, Bellucci, & Carlesimo, 2000), and others reporting reduced priming in ID individuals (Komatsu, Naito, & Fuke, 1996; Mattson & Riley, 1999). It was also reported that ID children showed significantly impaired procedural learning than TD children (Vicari, Bellucci, & Carlesimo, 2001). In the current study, we also observed a *reversed* priming effect when the same cue type was immediately repeated. These seemingly contradicting results could be explained by considering that implicit learning is not a unitary construct, and distinct neural circuits are implicated in repetition priming, perceptual-motor procedural learning, and operant conditioning (Barnes, Howard, Howard, Kenealy, & Vaidya, 2010; Squire, Stark, & Clark, 2004). For instance, Barnes et al. (2010) showed that ADHD children displayed atypical perceptual-motor sequence learning, but intact contextual learning, and explained the results that ADHD is mediated by their dysfunctional frontal-striatal-cerebellar circuits, which support implicit learning of perceptual-motor sequences but not visual-spatial context. Thus, it is reasonable to assume that dysfunctional history-driven attentional guidance in ID is observed in some forms of implicit learning and not in others, based on the etiology and neural circuits affected by ID. The literature review and theoretical explanations regarding the results in history-driven attentional mechanism has been added in the discussion section (p. 28-29).

4. Additional discussion of generalizability of findings should be included.

*Response.* We now have included discussion of generalizability of findings in the manuscript (p. 29).

**References**

Amso, D., Haas, S., Tenenbaum, E., Markant, J., & Sheinkopf, S. J. (2014). Bottom-Up Attention Orienting in Young Children with Autism. *Journal of Autism and Developmental Disorders, 44*(3), 664-673. doi:10.1007/s10803-013-1925-5

Barnes, K. A., Howard, J. H., Jr., Howard, D. V., Kenealy, L., & Vaidya, C. J. (2010). Two forms of implicit learning in childhood ADHD. *Dev Neuropsychol, 35*(5), 494-505. doi:10.1080/87565641.2010.494750

Bayliss, A., Di Pellegrino, G., & Tipper, S. (2005). Sex differences in eye gaze and symbolic cuing of attention. *The Quarterly journal of experimental psychology. A, Human experimental psychology, 58*, 631-650. doi:10.1080/02724980443000124

Bruyer, R., & Brysbaert, M. (2011). Combining speed and accuracy in cognitive psychology: Is the inverse efficiency score (IES) a better dependent variable than the mean reaction time (RT) and the percentage of errors (PE)? *Psychologica Belgica, 51*(1), 5-13. doi:10.5334/pb-51-1-5

Bryson, S. E., Bradley, E. A., Thompson, A., & Wainwright, A. (2008). Prevalence of Autism among Adolescents with Intellectual Disabilities. *The Canadian Journal of Psychiatry, 53*(7), 449-459. doi:10.1177/070674370805300710

Carlisle, N. B., Arita, J. T., Pardo, D., & Woodman, G. F. (2011). Attentional templates in visual working memory. *J Neurosci, 31*(25), 9315-9322. doi:10.1523/jneurosci.1097-11.2011

Chica, A. B., Martín-Arévalo, E., Botta, F., & Lupiáñez, J. (2014). The Spatial Orienting paradigm: How to design and interpret spatial attention experiments. *Neuroscience & Biobehavioral Reviews, 40*, 35-51. doi:<https://doi.org/10.1016/j.neubiorev.2014.01.002>

Chiviacowsky, S., Wulf, G., & Avila, L. T. (2013). An external focus of attention enhances motor learning in children with intellectual disabilities. *J Intellect Disabil Res, 57*(7), 627-634. doi:10.1111/j.1365-2788.2012.01569.x

Cohen, J. (2013). Statistical Power Analysis for the Behavioral Sciences.

Dodd, M. D., & Wilson, D. (2009). Training attention: Interactions between central cues and reflexive attention. *Visual cognition, 17*(5), 736-754. doi:10.1080/13506280802340711

Driver, J., Davis, G., Ricciardelli, P., Kidd, P., Maxwell, E., & Baron-Cohen, S. (1999). Gaze Perception Triggers Reflexive Visuospatial Orienting. *Visual cognition, 6*(5), 509-540. doi:10.1080/135062899394920

Einfeld, S. L., Ellis, L. A., & Emerson, E. (2011). Comorbidity of intellectual disability and mental disorder in children and adolescents: a systematic review. *J Intellect Dev Disabil, 36*(2), 137-143. doi:10.1080/13668250.2011.572548

Greenaway, R., & Plaisted, K. (2005). Top-Down Attentional Modulation in Autistic Spectrum Disorders Is Stimulus-Specific. *Psychological Science, 16*(12), 987-994. doi:10.1111/j.1467-9280.2005.01648.x

Hommel, B., Pratt, J., Colzato, L., & Godijn, R. (2001). Symbolic control of visual attention. *Psychol Sci, 12*(5), 360-365. doi:10.1111/1467-9280.00367

Kennett, S., Eimer, M., Spence, C., & Driver, J. (2001). Tactile-visual links in exogenous spatial attention under different postures: convergent evidence from psychophysics and ERPs. *J Cogn Neurosci, 13*(4), 462-478. doi:10.1162/08989290152001899

Komatsu, S.-i., Naito, M., & Fuke, T. (1996). Age-Related and Intelligence-Related Differences in Implicit Memory: Effects of Generation on a Word-Fragment Completion Test. *J Exp Child Psychol, 62*(2), 151-172. doi:<https://doi.org/10.1006/jecp.1996.0026>

Kuefner, D., Cassia, V. M., Vescovo, E., & Picozzi, M. (2010). Natural experience acquired in adulthood enhances holistic processing of other-age faces. *Visual cognition, 18*(1), 11-25. doi:10.1080/13506280802396507

La Malfa, G., Lassi, S., Bertelli, M., Salvini, R., & Placidi, G. F. (2004). Autism and intellectual disability: a study of prevalence on a sample of the Italian population. *J Intellect Disabil Res, 48*(Pt 3), 262-267. doi:10.1111/j.1365-2788.2003.00567.x

Langton, S. R. H., & Bruce, V. (2000). You must see the point: Automatic processing of cues to the direction of social attention. *Journal of Experimental Psychology: Human Perception and Performance, 26*(2), 747-757. doi:10.1037/0096-1523.26.2.747

Lowe, K., Allen, D., Jones, E., Brophy, S., Moore, K., & James, W. (2007). Challenging behaviours: prevalence and topographies. *Journal of Intellectual Disability Research, 51*(8), 625-636. doi:<https://doi.org/10.1111/j.1365-2788.2006.00948.x>

Marotta, A., Lupiáñez, J., Martella, D., & Casagrande, M. (2012). Eye gaze versus arrows as spatial cues: two qualitatively different modes of attentional selection. *J Exp Psychol Hum Percept Perform, 38*(2), 326-335. doi:10.1037/a0023959

Matson, J. L., & Shoemaker, M. (2009). Intellectual disability and its relationship to autism spectrum disorders. *Res Dev Disabil, 30*(6), 1107-1114. doi:10.1016/j.ridd.2009.06.003

Mattson, S. N., & Riley, E. P. (1999). Implicit and explicit memory functioning in children with heavy prenatal alcohol exposure. *J Int Neuropsychol Soc, 5*(5), 462-471. doi:10.1017/s1355617799555082

Merrill, E. C., Conners, F. A., Yang, Y., & Weathington, D. (2014). The acquisition of contextual cueing effects by persons with and without intellectual disability. *Res Dev Disabil, 35*(10), 2341-2351. doi:10.1016/j.ridd.2014.05.026

Posner, M. I. (1980). Orienting of attention. *Quarterly Journal of Experimental Psychology, 32*, 3-25.

Reber, A. S. (1992). The cognitive unconscious: An evolutionary perspective. *Consciousness and Cognition, 1*(2), 93-133. doi:<https://doi.org/10.1016/1053-8100(92)90051-B>

Ristic, J., & Kingstone, A. (2006). Attention to Arrows: Pointing to a New Direction. *Quarterly Journal of Experimental Psychology, 59*(11), 1921-1930. doi:10.1080/17470210500416367

Saffran, J. R. (2018). Statistical learning as a window into developmental disabilities. *Journal of Neurodevelopmental Disorders, 10*(1), 35. doi:10.1186/s11689-018-9252-y

Smith, K. R., & Matson, J. L. (2010). Behavior problems: differences among intellectually disabled adults with co-morbid autism spectrum disorders and epilepsy. *Res Dev Disabil, 31*(5), 1062-1069. doi:10.1016/j.ridd.2010.04.003

Squire, L. R., Stark, C. E. L., & Clark, R. E. (2004). THE MEDIAL TEMPORAL LOBE. *Annual Review of Neuroscience, 27*(1), 279-306. doi:10.1146/annurev.neuro.27.070203.144130

Vicari, S., Bellucci, S., & Carlesimo, G. A. (2000). Implicit and explicit memory: a functional dissociation in persons with Down syndrome. *Neuropsychologia, 38*(3), 240-251. doi:10.1016/s0028-3932(99)00081-0

Vicari, S., Bellucci, S., & Carlesimo, G. A. (2001). Procedural learning deficit in children with Williams syndrome. *Neuropsychologia, 39*(7), 665-677. doi:10.1016/s0028-3932(01)00012-4

Woodman, G. F., Luck, S. J., & Schall, J. D. (2007). The role of working memory representations in the control of attention. *Cerebral Cortex, 17*(Suppl 1), 118-124. doi:10.1093/cercor/bhm065

Wyatt, B. S., & Conners, F. A. (1998). Implicit and explicit memory in individuals with mental retardation. *Am J Ment Retard, 102*(5), 511-526. doi:10.1352/0895-8017(1998)102<0511:iaemii>2.0.co;2
