# Supplementary material for: Top-down, bottom-up, and history-driven processing of multisensory attentional cues in intellectual disability: An experimental study in virtual reality
Source: PLoS One. 2021 Dec 21;16(12):e0261298. doi: 10.1371/journal.pone.0261298 (PMC8691646; doi:10.1371/journal.pone.0261298)
Supplement: Attachment — Submitted filename: Response to Reviewers.docx [file pone.0261298.s003.docx]

**Reviewer 1**

Previous point 1. If they showed such prominent orienting during practice, it is puzzling that they showed no cuing effect for those non-visual endogenous cues in the main experiment. It is also unclear how long these rules were retained. Given the obvious difference in the need to retain an active set of rules in WM for the endogenous (non-visual) cues and the difficulty that ID participants would be expected to have with this by definition, I found this initial practice session an underwhelming basis upon which to completely rule out the idea that the results could be at least partly explained by an impaired ability to closely follow task rules. I think a sterner caveat would be appropriate here.

*Response.* The core feature that defines “endogenous” cues is that they are only *symbolic* of the target location, and therefore inherently require *learning* of the arbitrary rules that associate each symbol with a particular location. In that sense, learning and retaining the associative rules in working memory and utilizing that knowledge to deploy attention accordingly are both critical cognitive components that determine the individual’s top-down attentional performance. Not only space-based attention, but also feature-based or object-based attention relies heavily on the WM function: A consensus among theories of attention is that the contents of the “attentional template”, the collection of task-relevant features held in working memory (Bundesen, 1990; Carlisle, Arita, Pardo, & Woodman, 2011), determine the efficiency of voluntary attentional allocation by adjusting sensory gain to distinguish target features from nontarget features (Hout & Goldinger, 2015; Martinez-Trujillo & Treue, 2004; Wolfe, 1994). Considering that maintaining the precise attentional template in WM and utilizing that information to deploy cognitive resources are both required for successful top-down attentional performance, we do not believe that the possibility of impaired WM function in the ID group should be completely ruled out in order to show impaired top-down attentional performance, as the reviewer suggested. Rather, the imprecise attentional template in WM could be one of the reasons behind the diminished top-down attentional performance in the ID group. The followings are several possible reasons behind the diminished effect of endogenous cues in the ID group: i) ID participants did not learn the meaning of endogenous cues at all. This is not likely, since instruction and practice sessions were repeated until all experimenters agreed upon the participant’s successful understanding of the meaning of each cue type. ii) ID participants did learn the meaning of endogenous cues, but the memory trace was weaker or decayed faster, making the attentional template in WM imprecise. iii) ID participants successfully learned and retained the associative rules, but did not utilize the knowledge to deploy attention for simplicity or conservation of cognitive resources. iv) ID participants did learn and retain the associative rules, but had impaired function of deploying cognitive resources according to the knowledge. v) combination of above reasons in various degrees. Elucidating the extent of each cognitive process’ involvement in producing the reduced effect of endogenous cues would be an important and interesting topic for future studies, and we now have added the idea in the discussion section of the manuscript (p. 29).

Previous point 2. I appreciate those additions to the text, but in the abstract and elsewhere (e.g., Results), the authors still make the claim that how ID affects attention is specific to certain sensory modalities which may not be the case. I would recommend more carefully tempering those claims (especially in the abstract, which lacks this important context).

*Response.* Following the reviewer’s suggestion, we now have revised sentences in the abstract (p. 2) and results section (p. 21) in the manuscript, such that the differences in cueing effects across cue types are simply described, rather than emphasizing that the impact of ID on attentional processing is specific to certain sensory modalities.

Previous point 3. I still do not understand how a 100% valid cue can be called an exogenous cue (regardless of whether it is compared to a neutral cue). The cue is highly informative. I think the authors need to be more cautious with the terminology they use here: many readers will see "exogenous" and assume reflexive orienting to a non-predictive cue, and without the cue being non-predictive, there is no way to know just how exogenous the associated orienting response is. That the authors call these exogenous cues is really just an assumption, and although I agree that these cues likely have some exogenous component, for all we know the cueing effects associated with them could be largely dominated by endogenous processes (calling into question that more-or-less categorical distinction forwarded in the paper and reified in the labeling of the task conditions). Indeed, they are just as informative as the endogenous cues (100%). I realize it is convenient to have an endogenous/exogenous factor and label it as such, but I do not think that is appropriate here.

*Response.* We understand the reviewer’s concern about the informativeness of our exogenous cues, and acknowledge that making them 100% valid was not ideal for pinpointing only the reflexive component of orienting. As we already explained in the previous round of revision, we included neutral cues, instead of invalid cues, since including invalid trials and manipulating the probability between valid and invalid trials should involve statistical learning abilities, which are reported to be significantly different between TD and ID groups (Saffran, 2018), and therefore would confound the results. Moreover, even if we made the exogenous cues noninformative by having 50% invalid and 50% valid trials, it alone cannot guarantee that the associated orienting response is purely reflexive: Participants could still show an endogenous bias towards the cued side, since there is no harm in strategically prioritizing the location that already captured attention by an abrupt onset or a salient feature. Making it harmful to prioritize the cued side by increasing the probability of invalid trials would also be problematic, since it should involve top-down suppression mechanisms. Thus, non-predictivity of cues does not ensure that the effect is 100% reflexive. Although we agree with the reviewer that non-predictivity is one of important features that accompany exogenous cues, we believe that abrupt onset, saliency, and peripheral location are core elements that distinguish exogenous cues from endogenous cues (symbolic, non-salient, central location), all of which were applied to the cue types in the current study.

Previous point 4. I appreciate that there are not many trials per cell, but I am not sure what exactly this analysis gets at without going into greater depth. What exactly does a mere repetition of cue type (collapsed across all other factors) tell you about selection history? And I understand that the "examination of the repetition priming effect was independent from the cueing effect analyses," but I still do not see how the lack of cueing effect does not call the meaningfulness of this analysis into question to some degree. If there is no evidence that ID participants "selected" the cue in some cases, then how can this analysis speak to "selection history" in those cases? What is the attentional process that the authors hypothesize is repeating in this case that could be blunted or accentuated by ID?

*Response.* In the current study, the efficiency of history-driven attentional mechanism was examined in three different ways: i) *cue type* repetitions regardless of target side or location, ii) *target-side* repetitions regardless of the exact target location, iii) exact *target-location* repetitions. The latter two analyses revealed that both TD and ID groups showed the target-side and target-location repetition priming effect in a similar way, suggesting that the function of the history-driven attentional mechanism that guides attention to the previously selected *target in space* is preserved in ID (p. 24-25). On the other hand, the cue type repetition analysis examined the effect of selecting and utilizing the cue information to deploy attention, regardless of the spatial location of the upcoming target. That is, if the history of encountering a specific cue type had a significant priming effect, then the cue would be selected and utilized more to guide attention in trials where the same cue type is immediately repeated. For instance, regardless of whether the tactile endogenous cue had a small or no cueing effect in one encounter, the degree of utilizing the tactile endogenous cue to guide attention might increase, by encountering the same cue type again in the immediate next trial. Consistently, the TD group showed better search performance in cue type-repeated trials than in non-repeated trials, showing significant cue type repetition priming effect. On the other hand, the ID group showed significant *decrease in search performance* in cue type-repeated trials as compared to non-repeated trials (p. 23). These results suggest that the function of history-driven attentional mechanism that enhances the processing of *repeated features of cue* is not only deteriorated but even reversed in individuals with intellectual disability. We now have added a sentence that clarifies the purpose of the cue type repetition analysis in the manuscript (p. 23).

Previous point 5: I understand that the WM burden of keeping the digit in active memory is in and of itself low, but this is coupled with the need to keep the rules for interpreting the different cues in active memory as well (which I assume was not the case in those Woodman et al. and like studies), and this just adds one more thing to remember that may be more likely to push ID participants over the edge of what they can keep in active memory. The concern is not that ID participants forget the target digit, which I agree would be inconsistent with measured accuracy, but rather that the combined demand caused some ID participants to forget the rules for interpreting the cue (much more so than control participants), leading to the lack of cueing effects in some cases. I still think this is a weakness of the paradigm that requires more careful consideration.

*Response.* Following the reviewer’s suggestion, we now have added sentences that consider the possibility of the working memory burden causing ID participants’ failures in utilizing certain cue types, as well as ideas for future studies with a fewer number of well-learned endogenous cues and a fixed search target to lower the burden on working memory in the discussion section of the manuscript (p. 28).

Previous point 6: With such a small sample size, it is not much to show that the samples did not significantly differ on characteristics like gender. Only a very large imbalance will show up as significant. There are literally almost twice as many females in the TD group (!!), and this could be at least to some degree influencing the results. The TD sample is just university students, so I still do not understand why the samples were not more closely matched, especially with respect to gender. This at least deserves a sterner caveat.

*Response.* We agree with the reviewer that completely matching the gender, age, and other demographic characteristics of the participants in ID and TD group would have been ideal. To explain more about our sampling procedure, we first recruited the ID group participants according to our inclusion and exclusion criteria at a developmental disability training center, and then recruited TD participants at a university trying to balance the demographic characteristics between two groups. However, the age range of the ID group was 22 to 50 years (M = 30.6, SD = 7.26), which was higher than that of typical university students. Thus, in order to balance the chronological age of the two groups, we had to recruit older employees as well as students. Even though we did our best to match the age and gender of the two groups, it was not that easy to recruit 1:1 matched participants. To examine the possibility that gender of the participants might have differentially influenced attentional performance, we conducted our main analyses again with gender as a between-subject factor; The cue effect data were entered into a mixed ANOVA with sense (visual, auditory, tactile) and origin (endogenous, exogenous) of the cue as within-subject factors and gender (female, male) as a between-subject factor. The results revealed no significant main effect [*F*(1, 38) = .05, *p* = .82, $\eta_{p}^{2}$ = .001] or interactions [*F*s < 1.66, *p*s > .20, $\eta_{p}^{2}$s < .042] involving gender, supporting that gender did not differentially affected the search performance of participants. These results have now been added in the results section of the manuscript (p. 18) as well as in the supplementary file (p. 3).

**Reviewer 2**

1. The exploratory research questions should be stated (and justified) in the last paragraph of the introduction.

*Response.* Following the reviewer’s suggestion, sentences that introduce additional exploratory analyses have been added in the last paragraph of the introduction section (p. 8), in addition to the explanations added to the method section in the previous round of revision (p. 13, p. 15).

2. Effect sizes (including for non-significant results) should be presented for all analyses, including t tests.

*Response.* We now have added effect sizes (partial eta squared, Cohen’s d, or Cramer’s V) for all analyses (including non-significant results) throughout the manuscript and the supplementary file.

3. If sample size was based on the main RQ, it looks that some exploratory RQ are underpowered. Either being or not the case, this issue should be discussed in the manuscript.

*Response.* Caveats regarding the sample size and underpower have been added in the discussion section of the manuscript (p. 31).

4. As a side note, the submission guidelines present lowercase p-values: “P-values. Report exact p-values for all values greater than or equal to 0.001. P-values less than 0.001 may be expressed as p < 0.001, or as exponentials in studies of genetic associations.

*Response.* Lowercase p is now used for reporting p-values throughout the manuscript and the supplementary file.

**References**

Bundesen, C. (1990). A theory of visual attention. *Psychological Review, 97*(4), 523-547.

Carlisle, N. B., Arita, J. T., Pardo, D., & Woodman, G. F. (2011). Attentional templates in visual working memory. *J Neurosci, 31*(25), 9315-9322. doi:10.1523/jneurosci.1097-11.2011

Hout, M. C., & Goldinger, S. D. (2015). Target templates: the precision of mental representations affects attentional guidance and decision-making in visual search. *Atten Percept Psychophys, 77*(1), 128-149. doi:10.3758/s13414-014-0764-6

Martinez-Trujillo, J. C., & Treue, S. (2004). Feature-based attention increases the selectivity of population responses in primate visual cortex. *Curr Biol, 14*(9), 744-751. doi:10.1016/j.cub.2004.04.028 S0960982204002684 [pii]

Saffran, J. R. (2018). Statistical learning as a window into developmental disabilities. *Journal of Neurodevelopmental Disorders, 10*(1), 35. doi:10.1186/s11689-018-9252-y

Wolfe, J. M. (1994). Guided Search 2.0: A revised model of visual search. *Psychonomic bulletin & review, 1*(202-238).
